# Ectopic cervical thymoma in a patient with Myasthenia gravis

**DOI:** 10.1186/1749-8090-6-89

**Published:** 2011-07-06

**Authors:** Ti Hei Wu, Jong Shiaw Jin, Tsai Wang Huang, Hung Chang, Shih Chun Lee

**Affiliations:** 1 Division of Thoracic Surgery, Department of Surgery, Tri-Service General Hospital, National Defense Medical Center, Taipei, Taiwan; 2 Department of Pathology, Tri-Service General Hospital, National Defense Medical Center, Taipei, Taiwan

## Abstract

Ectopic cervical thymoma is rare and is often misdiagnosed as a thyroid tumor or other malignancy. Ectopic thymic tissue can be found along the entire thymic descent path during embryogenesis. However, a thymoma arising from such ectopic thymic tissue is extremely rare. Herein we report a patient with ectopic cervical thymoma and myasthenia gravis (MG) and discuss the management.

## Background

Ectopic cervical thymomas are very rare and often present as palpable neck masses. Ectopic cervical thymoma presenting in patients with MG is even rarer and only two other cases have been reported in the literature [1,2]. The diagnosis is very difficult to make and has a major diagnostic pitfall. Extended thymectomy offered a good result for these patients. Herein we present a case of ectopic cervical thymoma associated with MG.

## Case report

**Figure 1:**
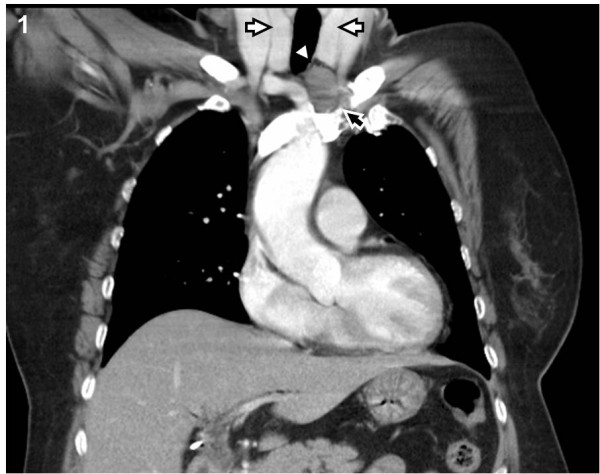
**Contrast-enhanced computed tomography of the chest showed a tumor mass (black arrow) at the lower pole of the left thyroid gland (white arrow)**. The fat plane (arrowhead) between the thyroid gland and the tumor was clear.

**Figure 2:**
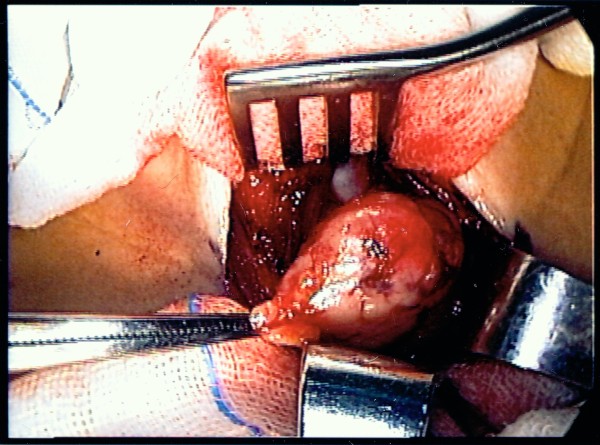
**Photography of transcervical thymomectomy illustrating one well-encapsulated tumor located between the upper pole of the left thymus gland and lower pole of the left thyroid gland**.

**Figure 3:**
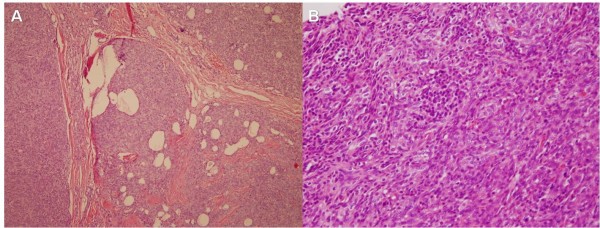
**Photomicrographys (hematoxylin and eosin staining)**. (A) Lobules separated by thick fibrous bands (× 40). (B) Microscopic findings of transition between lymphocyte-rich area and the lymphocyte-poor oval or spindle-shaped epithelial cell components, compatible with a WHO type AB thymoma (× 400).

A 58-year-old woman presented to our Neurology department with ptosis that had persisted for 4 months. A physical examination revealed a palpable cervical mass. Repetitive nerve stimulation testing revealed abnormally decreasing responses and the acetylcholine receptor antibody titer in the patient's serum was elevated (8.6 nmol/L, normal <0.2 nmol/L). Other laboratory examinations were unremarkable, including thyroid function tests and tests for autoimmune diseases. She was diagnosed with MG and received pyridostigmine treatment (180 mg/day). Computed tomography (CT) of her chest revealed one well- circumscribed, homogeneous mass of soft tissue measuring 2.6 × 2.5 × 1.6 cm at the lower pole of the left thyroid gland (Figure 1). The fat plane between the thyroid gland and the tumor was clear. The patient was subsequently referred to our thoracic surgery department and underwent extended transcervical thymomectomy and transsternal thymectomy. A well-encapsulated soft multi-lobulated tumor measuring 3 × 3 × 1.5 cm was found separately from the thoracic thymic gland, located between the upper pole of the left thymus gland and lower pole of the left thyroid gland (Figure 2). The cut surface of the tumor was tan-colored with no areas of necrosis. A total of 40 gm of thymic tissue was removed additionally. The anatomopathological examination of the sample using optical microscopy and immunohistochemical tests confirmed the diagnosis of an ectopic thymoma (Figure 3). The microscopy demonstrated the tumor comprised a mixture of lymphocyte-poor spindle cell areas and lymphocyte-rich areas. These histopathologic findings were consistent with a type AB according to World Health Organization Classification System (WHO), Masaoka stage I. The postoperative course was uneventful and the patient was discharged seven days after the operation. The patient was in complete remission at a three-month follow-up, and pharmacologic remission at a six-month follow-up.

## Discussion

The thymus is a lymphoepithelial organ that is derived embryologically from the third and fourth pharyngeal pouches, which descend to the anterior mediastinum in the sixth week of human gestation. Aberrant migration or remnants might occur along the entire path of thymic descent, and up to 20% of individuals are found to have these aberrant features [3]. Thymomas arising from aberrant thymic tissue are extremely rare, and the incidence of ectopic cervical thymoma is unknown. To the best of our knowledge, fewer than 30 cases of ectopic cervical thymoma have been published in the literature [1,4-17]. As mediastinal tumors, WHO type AB represent the most common subtype of ectopic cervical thymomas [5].

Patients with mediastinal thymomas are often clinically asymptomatic (50%-60%) or present as local symptoms (30% to 40%) or associated systemic parathymic disease syndromes (30% to 50%). When there are local symptoms, vague chest pain, shortness of breath, and cough are the common complaints. When there are systemic parathymic disease syndromes, MG is the most commonly associated disease (30%-50%) [18]. Relatively, ectopic cervical thymomas most commonly present as palpable neck masses and are misdiagnosed as thyroid masses. Only two other patients in the literature have presented with MG as the symptom [1,2]. The diagnosis of an ectopic cervical thymoma is very difficult to make and has a major diagnostic pitfall. As mentioned above, most patients present with a palpable neck mass and are misdiagnosed as having thyroid tumors. Further pathology, such as fine needle aspiration cytology, is needed to establish the nature of the so-called ''thyroid tumor''. Because the thymus gland is composed of epithelial and lymphoid elements, it could be misdiagnosed as a squamous cell carcinoma or lymphoma [7,10]. In our case, the patient presented with MG and the chest CT scan showed a clear fat plane between the cervical mass and the thyroid gland, which suggested that the cervical mass was separate from the thyroid. Therefore we thought the cervical mass was an ectopic thymoma and avoided tissue biopsy, opting for surgery.

Most ectopic cervical thymomas misdiagnosed as thyroid tumors were removed simply by a neck incision, because the exact diagnosis was made after postoperative histopathology. For ectopic cervical thymomas with MG, extended thymectomy seems to be the treatment of choice, like mediastinal thymomas. Of the two other case reports of ectopic cervical thymoma with MG in the literature, one received extended thymectomy and the other received simple resection of the ectopic cervical thymoma. The one received extended thymectomy achieved complete remission and the one received simple resection of the ectopic cervical thymoma achieved pharmacological remission during the long-term follow-up. Although our patient achieved only pharmacologic remission at a six-month follow-up, but the outcome of extended thymectomy improved gradually and took 3 years to achieve plateau [19]. Long-term follow-up of our patient is required to confirm the result more precisely. Overall, the outcomes of thymectomies for patients with MG and an ectopic cervical thymoma were good.

For ectopic thymomas with capsule invasion, adjuvant radiotherapy may be considered to reduce local recurrence rates as the general rule in mediastinal thymomas. However, the number of patients in this subgroup was limited, so more cases collection is required to confirm the result.

## Conclusion

Although the condition is rare, clinicians must bear in mind that ectopic cervical thymomas might be associated with MG. Extended thymectomy can offer a good result for these patients.

## Consent

Written informed consent was obtained from the patient for publication of this case report and accompanying images. A copy of the written consent is available for review by the Editor-in-Chief of this journal.

## Competing interests

The authors declare that they have no competing interests.

## Authors' contributions

THW carried out the manuscript and collected references. JSJ reported pathological findings and took the pathologic pictures. TWH and HC helped to draft the manuscript. THW and SCL underwent this operation. All authors read and approved the final manuscript.
